# Redefining the phenotype of alpha-methylacyl-CoA racemase (AMACR) deficiency

**DOI:** 10.1186/s13023-024-03358-9

**Published:** 2024-09-23

**Authors:** Femke C.C. Klouwer, Stefan D. Roosendaal, Carla E. M. Hollak, Mirjam Langeveld, Bwee Tien Poll-The, Arlette J. van Sorge, Nicole I. Wolf, Marjo S. van der Knaap, Marc Engelen

**Affiliations:** 1 Department of Pediatric Neurology, Emma Children’s Hospital, Amsterdam University Medical Centers, Amsterdam Leukodystrophy Center, Amsterdam, The Netherlands; 2 Department of Radiology, Amsterdam University Medical Centers, Amsterdam, The Netherlands; 3 Department of Endocrinology and Metabolism, and Amsterdam Gastroenterology Endocrinology and Metabolism research institute, Amsterdam University Medical Centers, Amsterdam, The Netherlands; 4 Department of Ophthalmology, Amsterdam University Medical Centers, Amsterdam, The Netherlands; 5 Amsterdam Neuroscience, Cellular & Molecular Mechanisms, Vrije Universiteit, Amsterdam, The Netherlands

**Keywords:** AMACR, Alpha-methylacyl-CoA racemase, Peroxisomal disorder, Phenotype, Bile acid synthesis

## Abstract

**Background:**

Alpha-methylacyl-CoA racemase (AMACR) deficiency is a rare peroxisomal enzyme deficiency caused by biallelic variants in the *AMACR* gene. This deficiency leads to the accumulation of toxic bile acid intermediates (*R*)-trihydroxycholestenoic acid (THCA) and (*R*)-dihydroxycholestenoic acid (DHCA) and pristanic acid. With less than 20 patients described in literature, the phenotype of AMACR deficiency is poorly defined and no data on the natural history are available.

**Results:**

Here we describe a cohort of 12 patients (9 adults and 3 children) with genetically confirmed AMACR deficiency (median age at diagnosis 56 years, range 3–69), followed for an average of 6 years (between 2015 and 2023). Five novel pathogenic variants are described. In 5/9 adult patients, retinitis pigmentosa was detected at a median age of 45 years (range 30–61). The median delay to diagnosis of AMACR deficiency after the diagnosis of retinitis pigmentosa was 24 years (range 0–33). All adult patients subsequently developed neurological signs and symptoms after the age of 40 years; most frequently neuropathy, ataxia and cognitive decline with prior normal cognitive functioning. One patient presented with a stroke-like episode. All adult patients showed a typical MRI pattern involving the thalami and gray matter structures of the pons and midbrain. One patient had a hepatocellular carcinoma at the time of the AMACR deficiency diagnosis and two patients suffered from gallstones. All three included children had elevated liver transaminases as single presenting sign and showed no brain MRI abnormalities.

**Conclusion:**

AMACR deficiency can be considered as an adult slowly progressive disease with a predominant neurological phenotype. The main signs comprise retinitis pigmentosa, neuropathy, ataxia and cognitive decline; stroke-like episodes may occur. Recognition of typical MRI abnormalities may facilitate prompt diagnosis. In addition, there is a risk of liver fibrosis/cirrhosis and hepatocellular carcinoma in these patients, requiring active monitoring.

## Background

**Fig. 1:**
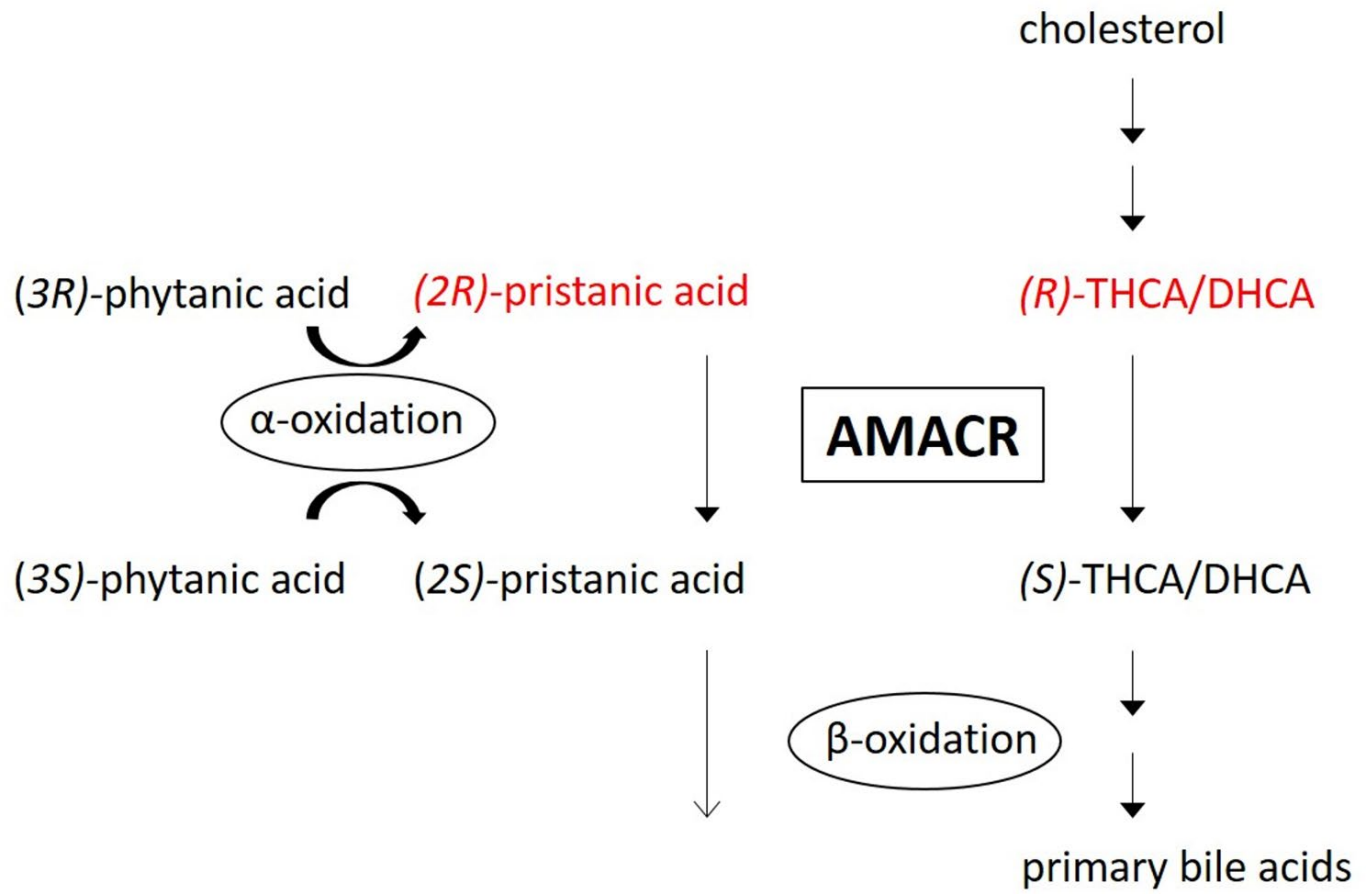
Schematic representation of the functions of alpha-methylacyl-CoA racemase (AMACR). The *(R)-*stereoisomers of pristanic acid, trihydroxycholestenoic acid (THCA) and dihydroxycholestenoic acid (DHCA) are converted to their corresponding *(S)-*isomer by AMACR. The *(S)-*isomers can be further processed by β-oxidation. Pristanic acid and phytanic acid are first converted to their corresponding CoA ester before oxidation (not shown here). Abnormal metabolites in case of AMACR deficiency are shown in red

Alpha-methylacyl-CoA racemase (AMACR) deficiency is an autosomal recessive peroxisomal disorder caused by pathogenetic variants in the *AMACR* gene that was first described in 2000 [1]. AMACR is responsible for the conversion of fatty acids with a methyl group in the (*R*)-configuration, including the branched-chain fatty acid pristanic acid and the C_27_-bile acid intermediates (*R*)- trihydroxycholestenoic acid (THCA) and (*R*)-dihydroxycholestenoic acid (DHCA), to the corresponding (*S*)-isomer. The (*S*)-isomers can be processed by peroxisomal β-oxidation. In the case of AMACR deficiency, bile acid intermediates (*R*)-DHCA and (*R*)-THCA and pristanic acid accumulate [1, 2] (Fig. 1). C_27_-bile acid intermediates are proven to be toxic, are associated with liver disease in multiple peroxisomal disorders, and are able to cross the blood-brain barrier with evidence of accumulation in brain in other peroxisome deficiency disorders with C_27_-bile acid intermediates accumulation [3, 4]. C_27_-bile acid intermediates are thought to be less capable than the primary C_24_-bile acids in forming mixed micelles in the small intestinal lumen, leading to malabsorption of fat and thereby of fat-soluble vitamins [5]. The diagnosis can be established by measuring the above mentioned metabolites in plasma, demonstration of C_27_- bile acid intermediates in urine, enzyme activity analysis in skin fibroblasts and mutation analysis of the *AMACR* gene (for review on the diagnosis of peroxisomal disorders and distinction between AMACR deficiency and other peroxisomal disorders see Klouwer et al. [6]).

To date, less than 20 patients with AMACR deficiency have been described in literature, mainly in separate case reports [1, 7–18]. One patient presented with neonatal cholestasis [14], most others were diagnosed in adulthood with presenting signs including retinitis pigmentosa, peripheral neuropathy, seizures, cognitive decline and encephalopathic and stroke-like episodes. Two patients had recurrent rhabdomyolysis [10, 16], and several children were diagnosed before development of symptoms because of incidental laboratory findings [13] or genetic testing for other indications [17]. No other patients than the neonate presenting with cholestasis were reported to suffer from liver disease, but one of the patients reported by Haugarvoll et al. [11] died with a liver tumor at age 51 years. Liver biopsy in this patient was suggestive of a liver sarcoma. Haugarvoll et al. also reported brain MRI findings in two adult patients with AMACR deficiency, who showed a similar pattern of T2-hyperintensities in thalami, midbrain and pons with gradual progression over time [11]. Overall, the phenotype of AMACR deficiency is poorly defined, and it is likely that only a part of the phenotypic spectrum is currently known. No data on the natural history of AMACR deficiency is available.

Here we describe the clinical, biochemical and genetic features of 12 patients with AMACR deficiency, the largest reported cohort of such patients to date. This study provides new insights in the spectrum of presenting signs and symptoms and tools for recognition of this rare neurometabolic disorder.

## Methods

### Patients

All patients with genetically confirmed AMACR deficiency who presented in the Amsterdam UMC (national expertise center for peroxisomal disorders) between 2015 and 2023 were included in this cohort. Approval of the Research Ethics Committee was not required since all measurements were performed as part of standard patient care. Written informed consent for publication was obtained from all patients or their legal representative.

### Biochemical analysis and liver stiffness measurements

Plasma bile acids were measured by using an ultra-high performance liquid chromatography tandem mass spectrometry method as described [19]. Urinary bile acids comprised primary bile acids (conjugates), bile alcohols and C_27_-bile acid intermediates and were measured qualitatively only [20]. Standard diagnostic assays were used to measure levels of conjugated bilirubin, plasma transaminases, vitamin D, E and A, and coagulation parameters. Liver stiffness analyses were performed using transient elastography (FibroScan^®^), according to the standard manufacturer instructions (Echosens, Paris, France) [21].

## Results

### Patient characteristics

A total of 12 patients (6 male, 6 female) were included in this cohort (Table 1). The median age at diagnosis was 56 years (range 3–69). All nine adult patients were symptomatic at diagnosis (median age at diagnosis 61 years, range 42–69); five adults were diagnosed after recognizing the typical brain MRI abnormalities, three adults were diagnosed by metabolic screening after MRI abnormalities gave rise to suspicion of a metabolic disorder and one adult was diagnosed after an older sibling was diagnosed with AMACR deficiency (patient 5). Three children were included (median age at diagnosis was 5 years, range 3–6). One child (patient 10) was diagnosed by metabolic screening because of the incidental finding of elevated liver enzymes in blood tests performed because of fatigue; one asymptomatic child (patient 12) was diagnosed following the diagnosis in an older sibling (patient 11), who underwent genetic analysis because of oculocutaneous albinism, which revealed a homozygous deletion of chromosome 5p13.3 encompassing five genes, including the *AMACR* gene [17]. The average time of follow-up was 6 years (range 1–9).

**Table 1:** Patient characteristics

Patient #	Sex	Allele 1 *AMACR* gene	Allele 2 *AMACR* gene	Age at diagnosis (years)	Follow-up (years)
**1**	Male	c.154T > C	c.154T > C	69	1
**2**	Male	c.155C > A	c.1040 dup	67	3
**3**	Male	c.364C > T	c.364C > T	66	3
**4**	Male	c.154T > C	c.154T > C	63	6
**5**	Female	c.364C > T	c.364C > T	61	5
**6**	Female	c.364C > T	c.364C > T	58	7
**7**	Female	c.154T > C	c.154T > C	53	9
**8**	Female	c.154T > C	c.364C > T	47	2
**9**	Female	c.154T > C	c.512G > A	42	5
**10**	Male	c.557A > G	c.557A > G	6	8
**11**	Male	5p13.3 del	5p13.3 del	5	9
**12**	Female	5p13.3 del	5p13.3 del	3	9

Most patients had homozygous missense variants in the *AMACR* gene (reference sequence NM_014324). Three patients were homozygous for the c.364 C > T (p.His122Tyr) variant, three patients were homozygous for the c.154T > C (p.Ser52Pro) variant and one patient homozygous for the c.557 A > G (p.Glu186Gly) variant. Two siblings had a deletion of the *AMACR* gene due to chromosome 5p13.3 deletion and three patient had compound heterozygous variants: c.154T > C (p.Ser52Pro) plus c.512G > A (p. Arg171His), c.154T > C (p.Ser52Pro) plus c.364 C > T (p.His122Tyr), and c.155 C > A (p.Ser52*) plus c.1040dup (p.Glu348Argfs*4). Only the c.154T > C (p.Ser52Pro) variant and chromosome 5p13.3 deletion have been previously reported [1, 17]. In all patients, AMACR deficiency was also confirmed by elevated plasma levels of pristanic acid and detection of C_27_- bile acid intermediates (*R*)-DHCA and (*R*)-THCA in blood.

### Clinical features

All adult patients presented with neurological signs that led to further investigations (Table 2). The most common signs among adult patients were sensomotor axonal neuropathy (*n* = 6/9), ataxia (*n* = 4/9) or cognitive decline with or without behavioral problems (*n* = 4/9). All these patients were above 40 years of age at the time of diagnosis. Patient 8 presented at the age of 46 years with subacute onset of headache, cognitive impairment, aphasia, apraxia and the development of right sided hemianopia over the course of several days. MRI of the brain showed no signs of recent ischemia, but did show symmetrical T2 hyperintense signal in the thalami and brain stem. EEG showed diffuse slowing of the left hemisphere. Partial spontaneous recovery occurred, but residual complaints of mild aphasia and hemianopia were reported. No possible triggering factor like preceding fasting or weight loss was identified in this patient. Two patients (patient 4 and 7) had a history of focal seizures (age 17 and 46 years respectively, at occurrence), which responded well to antiepileptic drugs. One patient (patient 7) was known to have intellectual impairment and suffered from an episode of depression with psychotic features. All other patients had normal cognition before cognitive or behavioral problems occurred. Five out of nine adult patients were previously diagnosed with retinitis pigmentosa with a median age at diagnosis of 45 years (range 30–61), but most of them reported vision problems already years before. The median delay to diagnosis of AMACR deficiency after the diagnosis of retinitis pigmentosa was 24 years (range 0–33). No patients had suffered from episodes of rhabdomyolysis or encephalopathic episodes other than the above described stroke-like episode.

**Table 2:** Signs at presentation and during follow-up

Organ system	Sign	n at presentation (total *n* = 12)	n during follow up (total *n* = 12)	Total (*n*, %)	Remarks
Nervous system	Cognitive decline	4	0	4/12 (33)	
	Intellectual impairment	1	N/A	1/12 (8)	
	Ataxia	4	1	5/12 (42)	
	Neuropathy	6	0	6/12 (50)	
	Dysphagia	1	1	2/12 (17)	
	Epilepsy	2	0	2/12 (17)	In one patient decades before diagnosis
	Psychiatric symptoms	1	0	1/12 (8)	
	Stroke-like episode	1	0	1/12 (8)	
Vision	Retinitis pigmentosa	5	0	5/12 (42)	
	Cataract	2	0	2/12 (17)	
	Glaucoma	1	0	1/12 (8)	
Gastrointestinal	Liver fibrosis	1	0	1/12 (8)	
	Oesophageal varices	0	1	1/12 (8)	
	Liver tumor	1	0	1/12 (8)	Multifocal hepatocellular carcinoma, one (unreported) sibling diseased of liver cancer
	Gallstones	2	0	2/12 (17)	
Other	Coagulopathy (symptomatic)	0	1	1/12 (8)	During treatment for malignancy
	Other malignancies	1	1	2/12 (17)	Melanoma, papillar urothelial carcinoma

*Abbreviations* n: number of patients, N/A: not applicable

One patient was diagnosed with liver fibrosis (patient 3), while all patients underwent regular abdominal ultrasounds and in most cases FibroScan^®^. In patient 3, ultrasound and MRI of the liver showed multifocal tumors in both liver lobes. The ultrasound was performed as part of regular work-up shortly after he was diagnosed with AMACR deficiency at the age of 66 years. Biopsy showed hepatocellular carcinoma and he died six months later of liver failure. Another patient had a sibling who died of liver cancer, who was not evaluated for AMACR deficiency at the time. Two patients had a history of cholecystectomy due to gallstones (patient 4 and 6, respectively around age 40 and 15 years). No patients were reported to have suffered from neonatal cholestasis. Other malignancies reported were ocular melanoma (patient 6) and urothelial carcinoma (patient 9), the latter diagnosed during follow-up.

Most patients did not develop any new disease signs during follow-up (see Table 2). All three children (patients 10–12) were asymptomatic at diagnosis and did not develop any signs during follow-up of at least seven years.

### Imaging

Magnetic resonance imaging (MRI) of the brain was performed in all patients. Contrast-enhanced images were available in four patients. The three children who underwent brain MRI (patient 10–12) between ages of 7 and 8 years showed no T2 hyperintensities, nor any other abnormalities. The age at the first performed MRI in the adult patients ranged between 35 and 67 years.

**Fig. 2:**
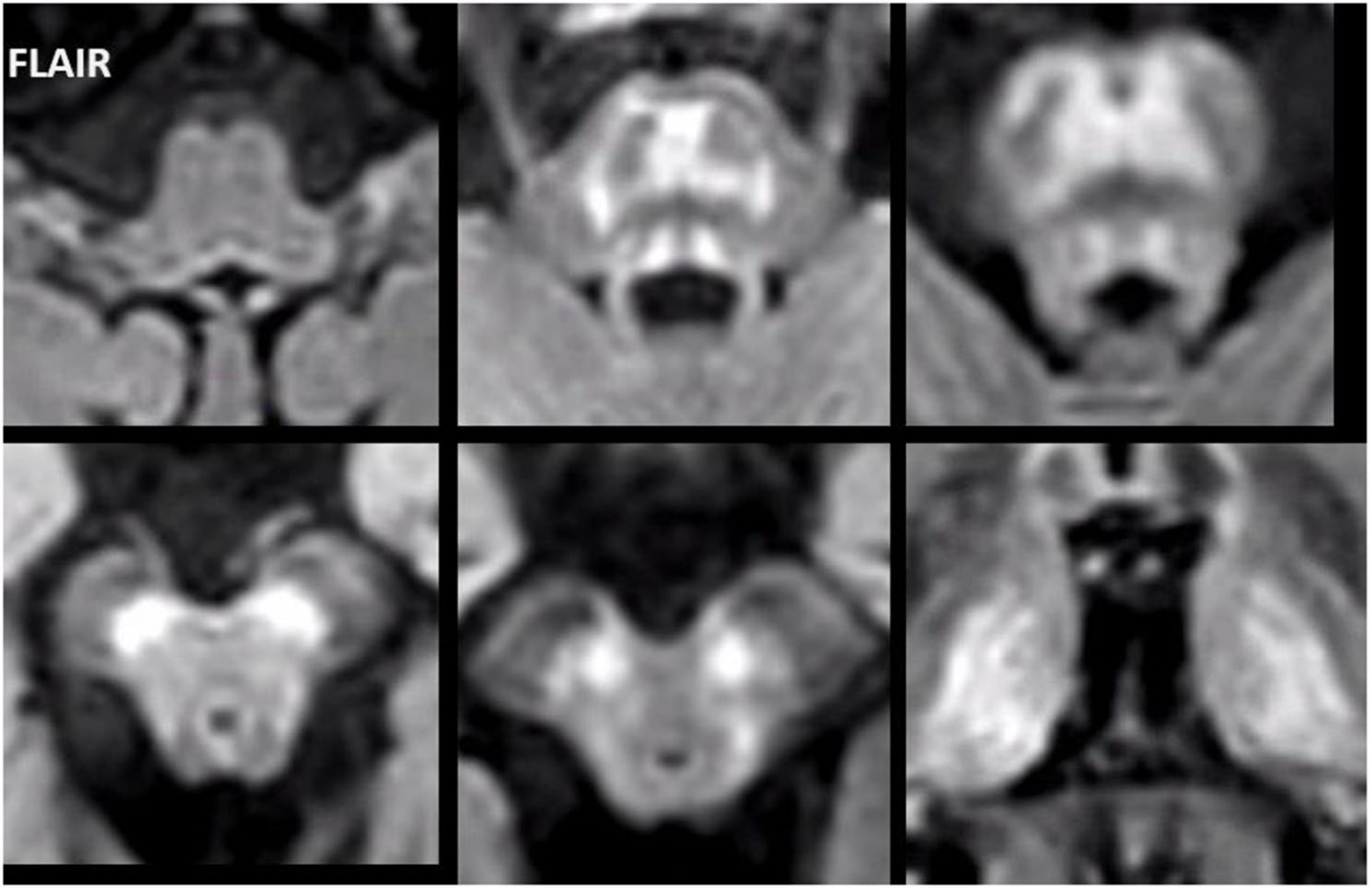
FLAIR images of patient 4 show a normal appearance of the medulla oblongata. The signal abnormalities of the pons are continuous with the affected substantia nigra. The red nucleus and thalamus are also symmetrically affected

**Fig. 3:**
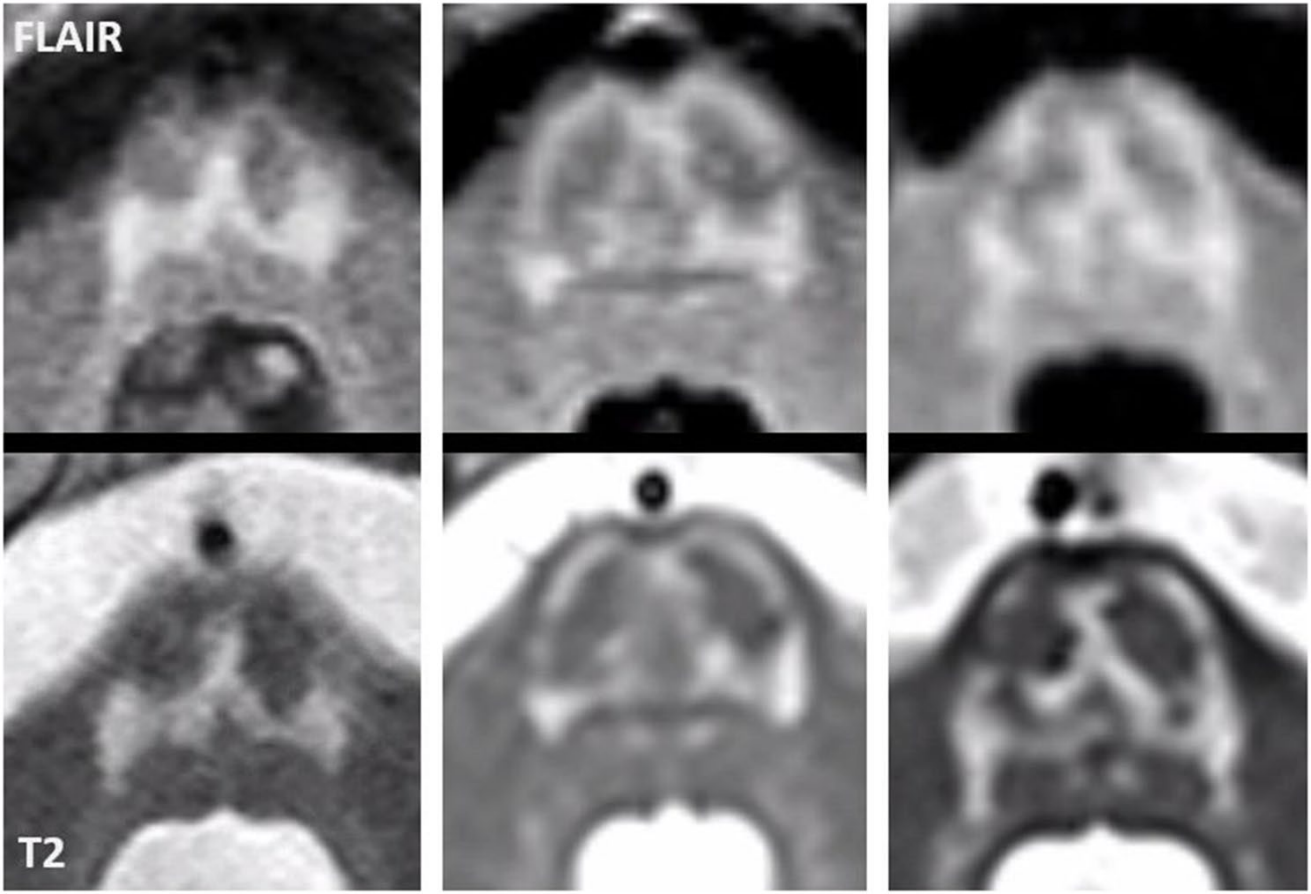
Cropped FLAIR and T2-weighted images of three different patients (from left to right: patient 3, 9 and 5) are shown, centered on the pons. Typical pontine gray matter involvement can be appreciated on both sequences, despite variation in quality of the images. Notice the sparing of the corticospinal tracts and the ascending sensory tracts, among which the medial lemniscus

In all adult patients, the first MRI already showed characteristic abnormalities. The typical MRI pattern consisted of symmetric T2 hyperintense signal abnormalities in the thalami, midbrain and pons (Fig. 2). The thalami were affected in eight of the nine patients. The midbrain was affected in all nine adult patients, including T2 hyperintensity of the substantia nigra in all patients and superior colliculi in seven patients. The grey matter of the pons was selectively involved in eight patients, sparing the corticospinal tracts as well as the ascending sensory tracts. This created either a trident-like configuration or a closed omega (ɷ) (Fig. 3), which was noted in four and two patients, respectively.

The medulla oblongata, middle cerebellar peduncles and cerebellar white matter showed no signal changes. Normally, the T2 signal of the dentate nucleus and the red nucleus is in general lower than the adjacent white matter in adults over 40 years of age. This was the case in none of our adult patients. The T2 signal of the dentate nucleus and red nucleus was evidently higher than white matter in three patients and five patients, respectively, and isointense with the surroundings in the other adult patients. The superior cerebellar peduncles exhibited subtly increased T2 signal in four patients.

**Fig. 4:**
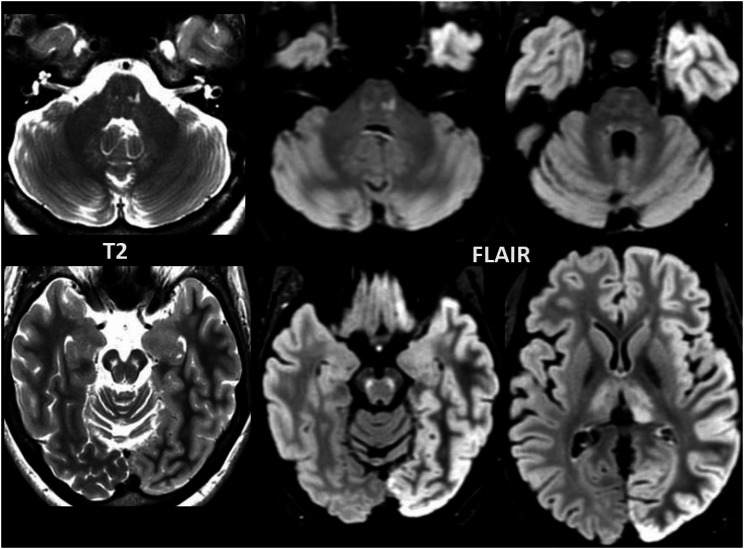
T2-weighted and FLAIR images of patient 8 are shown here. She presented with a (transient) stroke-like episode of aphasia, apraxia and hemianopsia, reflected by the cortical hyperintensity and swelling of the left cerebral hemisphere and reactive hyperintensity of the left pulvinar. The abnormalities in the pons are subtle and punctate in this patient, additionally the signal of the dentate nucleus is abnormal. The red nucleus and thalamus are involved

No abnormal contrast enhancement and no diffusion restriction were observed. In four patients atrophy of the brain stem disproportionate for age was noted, particularly of the midbrain and superior cerebellar peduncles. In patient 8, who presented with a stroke-like episode, the MRI showed cortical FLAIR hyperintensity and subtle cortical swelling of the left cerebral hemisphere, and presumably reactive hyperintensity of the left pulvinar (Fig. 4), in addition to AMACR-related abnormalities in the brain stem and thalami. In three patients, multifocal T2 hyperintensities of presumed vascular origin were observed in the cerebral white matter (patient 2, 6 and 7 at age 66, 62 and 52 years, respectively). In another patient confluent presumably vascular T2 hyperintensities were found in the cerebral white matter together with lacunar infarcts, small vermis infarcts and a cortical infarct (patient 4 at age 66).

Follow-up MRI of the brain with an interval greater than three years was available in four adult patients and one child. Three of the adults showed unchanged MRI abnormalities. Patient 4 underwent MRI with 29-year interval (age 37 and 66) and showed new periventricular white matter abnormalities, and progression of T2 hyperintensities at follow-up. In the child with follow-up MRI after 5 years still no abnormalities were found. In five out of the nine adult patients, the typical MRI abnormalities eventually led to the diagnosis. However, in multiple cases there was a delay of several years before the patient was referred and the already present MRI abnormalities were recognized as fitting with the above-described pattern seen in AMACR deficiency.

### Biochemical results

**Fig. 5:**
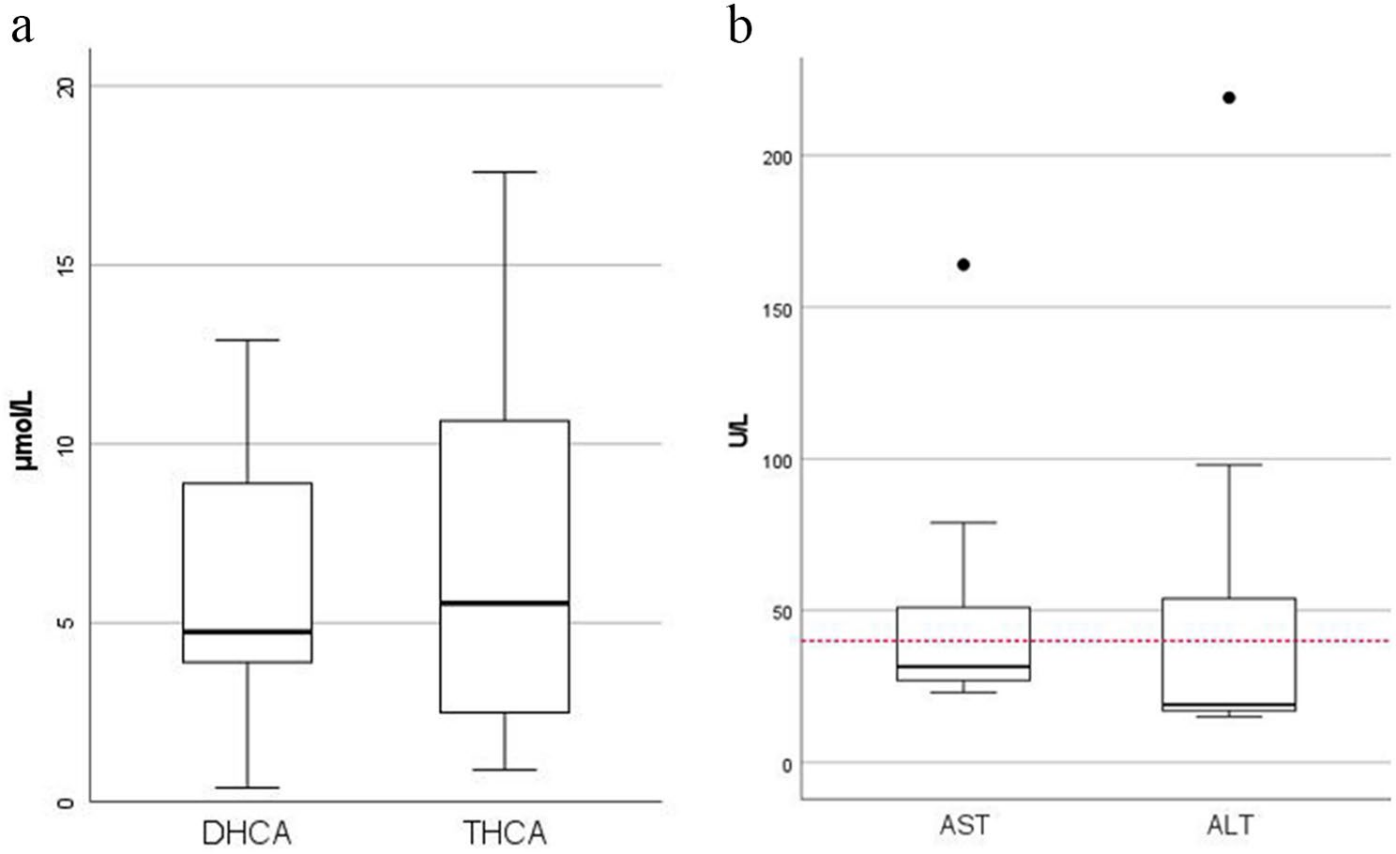
**A**. Boxplot of (*R*)-dihydroxycholestenoic acid (DHCA) and (*R*)-trihydroxycholestenoic acid (THCA) in all patients (*n* = 12). The upper reference range for DHCA is 0.0 µmol/L and 0.1 µmol/L for THCA. The median is shown as a solid line. **B.** Boxplot of aspartate aminotransferase (AST) and alanine aminotransferase (ALT) in 11 patients. The upper reference range (40 U/L) is indicated by the dotted horizontal line, the median as a solid line. Outliers are shown as black dots

Bile acid intermediates (R)-DHCA and (R)-THCA were elevated in all patients. The median DHCA level in plasma was 4.8 µmol/L (reference value 0.0 µmol/L) and THCA level in plasma was 5.6 µmol/L (reference value 0.0–0.1 µmol/L) (Fig. 5a). In one patient the primary bile acid cholic acid in plasma was below the lower limit of normal (reference value 0.1–4.7 µmol/L) and in five patients the primary bile acid chenodeoxycholic acid was below the lower limit of normal (reference value 0.7–10.0 µmol/L). In four patients, urinary bile acids were measured and C_27_-bile acid intermediates in urine were detectable in two of those patients. Pristanic acid was elevated in all patients (median 77.0 µmol/L, reference value 0.0–1.6 µmol/L). Phytanic acid was normal in six patients (median 9.5 µmol/L, reference value 0.49–9.88 µmol/L), but it was unclear whether some of these patients were already on a phytanic acid-restricted diet at the time of analysis.

Blood samples obtained after diagnosis, prior to possible vitamin supplementation, were analyzed for liver function tests, fat soluble vitamins and coagulation parameters. No coagulation parameters and vitamin levels were available for patient 1. Patient 3 was excluded from analysis of liver function tests because of liver cancer at the time of diagnosis. All 11 other patients had normal (total) bilirubin and alkaline phosphatase levels at the time of diagnosis. Only one adult had a slight elevated gamma-glutamyl transferase level in plasma. Five patients had elevated aspartate aminotransferase (AST) levels (median 51 U/L, range 46–146), three of whom are children. All three children also had elevated alanine aminotransferase (ALT) levels (54, 98 and 219 U/L respectively), which none of the adults had (Fig. 5b).

In six out of ten patients vitamin D deficiency was diagnosed, in three out of ten patients vitamin A deficiency and two out of ten patients vitamin E deficiency. Only one patient showed a slight prolonged prothrombin time (PT) and three patients showed a slight prolonged activated partial thromboplastin time (aPTT). Factor VII activity was measured in five patients, who all showed reduced activity (median 64%, reference value 80–140%), none of these patients showed clinical signs of a coagulopathy.

## Discussion

In this study, we characterized a cohort of 12 patients with AMACR deficiency. Two subgroups of patients could be identified in this cohort: adults who were diagnosed after developing clinical signs and who underwent brain MRI, which eventually led to the diagnosis, and clinically asymptomatic children who were diagnosed upon incidental findings or elevated liver enzymes and who remained asymptomatic during follow-up. Overall, a very similar clinical pattern could be observed in all adults: they may develop retinitis pigmentosa from young adulthood onwards without other guiding clinical signs; all subsequently develop neurological signs from the age of approximately 40 years onwards. Most common neurological signs in our cohort were sensomotor axonal neuropathy, ataxia and cognitive decline without a history of cognitive impairment. One patient suffered from a stroke-like episode, a phenomenon that has been previously described in AMACR deficiency [6], with unclear underlying pathophysiology. However, the high incidence of stroke-like episodes and transient encephalopathy with coma and seizures that was previously described by Tanti et al. [6] based on literature review of 16 patients with AMACR deficiency, was not observed in our cohort. Most patients remained clinically stable and developed no additional signs during years of follow-up, based on which AMACR deficiency can be considered a very slowly progressive disease.

We have performed a detailed analysis of the brain MRI abnormalities associated with AMACR deficiency. In the adult patients in our cohort the characteristic MRI pattern involved the pons, midbrain and thalami. Interestingly, we found that the structures involved in the brain stem are grey matter structures with sparing of the major white matter tracts, i.e., corticospinal tracts and ascending sensory tracts. This selective vulnerability is easier to appreciate by comparing MR images of patients with MR images of healthy adults acquired with a sequence optimized to discriminate brain stem nuclei [22]. On the FLAIR and T2-weighted images in most of our patients this grey matter pathology resulted in the pons in either a trident-like hyperintensity or a closed omega (ɷ). A trident-like abnormality in the pons can also be recognized in patients with osmotic demyelination syndrome [23], although in those cases it usually is more voluminous than in patients with an AMACR deficiency. Although MRI abnormalities in adults with AMACR deficiency have been described before [11], the pattern of selective grey matter vulnerability has not been appreciated previously. Recognition of this typical pattern facilitates prompt diagnosis. This is supported by the finding that all adults in our cohort showed characteristic MRI abnormalities at presentation and the majority was diagnosed after recognition of the typical pattern. The abnormalities involving the dentate nucleus, red nucleus and connecting dentatorubral tracts within the superior cerebellar peduncles, described earlier in a study by Haugarvoll et al. [11], are recognizable in part of our patient group. The signal changes in the dentate nucleus and superior cerebellar peduncles are generally less pronounced or more subtle than those in the pons, midbrain and thalamus. Since none of the children in our cohort showed MRI abnormalities at diagnosis or follow-up, it is unknown whether the neurological symptoms seen in the adult patients are preceded by MRI abnormalities and, if so, by how long. Also, we cannot rule out that the here reported children will have a different course of disease than the adults included in this cohort.

Although all three children had elevated liver transaminases (AST and ALT) levels, none of them had a history of cholestasis or showed any signs of clinically evident liver disease or fibrosis. Since also none of the adults in our cohort had a history of cholestasis, we conclude that the previous reported neonatal cholestasis in AMACR deficiency is most likely a very rare manifestation of disease or may have had a different cause in this particular patient [14]. One patient in our cohort had a hepatocellular carcinoma at the time the AMACR deficiency diagnosis was established and one (unreported) sibling of another patient who died of liver cancer was not evaluated for AMACR deficiency. Taking into account the patient reported by Haugarvoll et al. [11], who also developed a liver tumor, this underlines the need for awareness of the possibility of developing liver cancer in these patients and warrants screening for liver fibrosis and liver tumors in AMACR deficiency patients. It is known that liver cirrhosis predisposes to hepatocellular carcinoma independent of the underlying cause [24]. None of the patients in our cohort experienced rhabdomyolysis, so we cannot draw conclusions on this possible disease manifestation, except that this is most likely rare in AMACR deficiency. Both published patients with rhabdomyolysis were on antipsychotic medication [10, 16]. It remains therefore unknown whether AMACR deficiency can truly cause (recurrent) rhabdomyolysis or that these patients are more prone to a neuroleptic malignant syndrome due to neuroleptic medication.

There was a notable delay to diagnosis in the majority of the patients in our cohort while already multiple clinical signs or imaging findings could have been attributed to AMACR deficiency. It also raises the question whether a substantial proportion of AMACR deficiency patients still remains undiagnosed. Based on our data, targeted testing for AMACR deficiency should be considered in specific subgroups of patients to shorten this diagnostic delay. Since multiple of our patients had characteristic MRI abnormalities for years prior to diagnosis, increased awareness of the typical MRI pattern seen in AMACR deficiency is warranted. Screening should also be considered in young adults with retinitis pigmentosa without another identified cause, familial liver cancer or patients with liver fibrosis/cirrhosis or hepatocellular carcinoma without other risk factors. Considering the finding that all patients in our cohort had clearly elevated bile acid intermediates (DHCA and THCA) and pristanic acid in plasma independent of age, this is most likely a sensitive diagnostic tool for above-described purposes. We also plead for inclusion of the *AMACR* gene in existing gene panels for these disorders.

Given our data demonstrating that most patients with AMACR deficiency remain asymptomatic for the first decades of life, early diagnosis also provides the possibility of intervention with future potential therapies before the onset of some of the signs and symptoms. Cholic acid supplementation has been shown to suppress bile acid synthesis and thereby reduce the accumulation of toxic c_27_-bile acid intermediates in patients with a Zellweger spectrum disorder (i.e. a peroxisome biogenesis disorder), however, no improvement of clinically relevant parameters was observed [25]. Cholic acid supplementation has not been systematically studied in AMACR deficiency. Until specific therapeutic options are available, supportive management including dietary restriction of phytanic acid (i.e. to reduce pristanic acid accumulation), genetic and prognostic counselling should be offered to AMACR deficient patients.

Overall, our findings in the largest to date published cohort of AMACR patients contribute to a better understanding of the course of disease of AMACR deficiency, in which children may present with isolated elevated liver enzymes in the presence of abnormal peroxisomal metabolites (i.e. elevated C27 bile acid intermediates and pristanic acid) and adults develop a slowly progressive disease with a predominant neurological phenotype in combination with typical brain MRI abnormalities displaying selective grey matter vulnerability, resulting in a trident-like hyperintensity or a closed omega in the pons on the FLAIR and T2-weighted images. We also observed a general pattern in which patients first develop retinitis pigmentosa from young adulthood onwards and subsequently develop neurological signs after the age of 40 years; most often starting with ataxia and polyneuropathy, followed by cognitive decline. We confirmed that there is a potential risk of developing liver cancer in these patients. These findings lead to a better characterization of the phenotype of this rare disorder and provide tools for early diagnosis.

## Conclusions

Based on the characterization of 12 patients with AMACR deficiency in our cohort, AMACR deficiency can be considered as an adult slowly progressive disease with a predominant neurological phenotype. The main clinical signs comprise retinitis pigmentosa, neuropathy, ataxia and cognitive decline; stroke-like episodes may occur. Recognition of typical MRI abnormalities involving the brain stem and thalami may facilitate prompt diagnosis. Awareness of the risk of developing liver cancer in these patients is warranted.

## Data Availability

Additional data are available upon request.

## Abbreviations

ALT: Alanine aminotransferase
AMACR: Alpha-methylacyl-CoA racemase
aPTT: Activated partial thromboplastin time
AST: Aspartate aminotransferase
DHCA: Dihydroxycholestenoic acid
MRI: Magnetic resonance imaging
PT: Prothrombin time
THCA: Trihydroxycholestenoic acid
